# An Epigenomic Approach to Improving Response to Neoadjuvant Cisplatin Chemotherapy in Bladder Cancer

**DOI:** 10.3390/biom6030037

**Published:** 2016-09-02

**Authors:** Evanguelos Xylinas, Melanie R. Hassler, Dazhong Zhuang, Martin Krzywinski, Zeynep Erdem, Brian D. Robinson, Olivier Elemento, Thomas Clozel, Shahrokh F. Shariat

**Affiliations:** 1 Department of Urology, Weill Cornell Medical College, New York-Presbyterian Hospital, New York, NY 10065, USA; evanguelosxylinas@hotmail.com (E.X.); dz100005@gmail.com (D.Z.); 2 Institut National de la Santé et de la Recherche médicale (Inserm) Unit U955 Eq07, CHU Henri Mondor, Créteil 94010, France; 3 Department of Urology, Medical University of Vienna, Vienna 1090, Austria; melanie.hassler@meduniwien.ac.at; 4 Canada’s Michael Smith Genome Sciences Center, Cancer Research Center, Vancouver, BC V5Z4S6, Canada; martink@bcgsc.ca; 5 Institute of Cancer Research, Department of Medicine 1, Medical University of Vienna, Vienna 1090, Austria; zeynep.erdem@meduniwien.ac.at; 6 Department of Pathology, Weill Cornell Medical College, New York-Presbyterian Hospital, New York, NY 10065, USA; brr2006@med.cornell.edu; 7 Division of Physiology and Biophysics, Weill Cornell Medical College, New York-Presbyterian Hospital, New York, NY 10065, USA; ole2001@med.cornell.edu; 8 Division of Medical Oncology, Weill Cornell Medical College, New York-Presbyterian Hospital, New York, NY 10065, USA; thomas.clozel@gmail.com; 9 Department of Urology, University of Texas, Southwestern Medical Center, Dallas, TX 75390, USA

**Keywords:** *HOXA9*, urinary bladder neoplasms, drug resistance, neoadjuvant therapy, decitabine

## Abstract

Bladder cancer is among the five most common cancers diagnosed in the Western world and causes significant mortality and morbidity rates in affected patients. Therapeutic options to treat the disease in advanced muscle-invasive bladder cancer (MIBC) include cystectomy and chemotherapy. Neoadjuvant cisplatin-based combination chemotherapy is effective in MIBC; however, it has not been widely adopted by the community. One reason is that many patients do not respond to neoadjuvant chemotherapy, and no biomarker currently exists to identify these patients. It is also not clear whether a strategy to sensitize chemoresistant patients may exist. We sought to identify cisplatin-resistance patterns in preclinical models of bladder cancer, and test whether treatment with the epigenetic modifier decitabine is able to sensitize cisplatin-resistant bladder cancer cell lines. Using a screening approach in cisplatin-resistant bladder cancer cell lines, we identified dysregulated genes by RNA sequencing (RNAseq) and DNA methylation assays. DNA methylation analysis of tumors from 18 patients receiving cisplatin-based chemotherapy was used to confirm in vitro results. Cisplatin-resistant bladder cancer cells were treated with decitabine to investigate epigenetic sensitization of resistant cell lines. Our results show that *HOXA9* promoter methylation status is associated with response to cisplatin-based chemotherapy in bladder cancer cell lines and in metastatic bladder cancer. Bladder cancer cells resistant to cisplatin chemotherapy can be sensitized to cisplatin by the DNA methylation inhibitor decitabine. Our data suggest that *HOXA9* promoter methylation could serve as potential predictive biomarker and decitabine might sensitize resistant tumors in patients receiving cisplatin-based chemotherapy.

## 1. Introduction

Bladder cancer is the second most common genitourinary cancer, with an estimated 74,000 new cases and 16,000 deaths per year in the USA [1]. Radical cystectomy is the mainstay therapy for muscle-invasive bladder cancer (MIBC) [2]. Nevertheless, up to 50% of patients experience disease recurrence despite seemingly effective local therapy. Level I evidence supports the use of neoadjuvant cisplatin-based combination chemotherapy in MIBC patients [3]. However, to date, neoadjuvant chemotherapy has not been adopted by the community and is less commonly used than adjuvant chemotherapy [4,5]. The reasons for these practice patterns are multifactorial, but apprehension regarding a possible resistance to a toxic chemotherapeutic drug remains one of the main reasons. Identification of those patients who will most benefit from cisplatin-based combination therapy, while avoiding toxicity and a delay in surgery from an ineffective therapy in those who are not likely to respond, would be very useful in daily clinical decision-making. Furthermore, non-toxic sensitization strategies for tumors may significantly improve outcome by lowering chemotherapeutic dosage and/or increasing cytotoxicity to tumor cells while limiting side effects. 

Chemoresistance is due to genetic and epigenetic alterations accumulating in response to treatment [6,7,8]. Whereas genetic mutations cannot be reversed, epigenetic alterations such as DNA methylation and histone modifications can be potentially reconfigured to a non-resistant state by the application of DNA methyltransferase (DNMT) and histone deacetylase (HDAC) inhibitors. The DNMT inhibitor decitabine and the HDAC inhibitor vorinostat have shown promising results regarding epigenetic sensitization in patients suffering from diffuse B-cell lymphoma, ovarian cancer and non-small cell lung cancer [9,10,11].

In a translational approach to study cisplatin-based chemotherapy resistance, we evaluated a panel of 35 bladder cancer cell lines for treatment-refractory patterns. We hypothesized that bladder cancer cell lines would show differential sensitivity responses to standard chemotherapeutic drugs. Furthermore, we expected that gene expression signatures of sensitive and resistant cells (pre-treatment) could help us to identify molecular markers associated with response to chemotherapeutic drugs. We identified a distinct gene signature associated with resistant cell lines that could predict response to cisplatin in our preclinical model. Among the gene signature, we found the homeobox gene *HOXA9* to be specifically downregulated by promoter DNA hypermethylation in resistant cell lines. We confirmed *HOXA9* promoter methylation status in both cell lines and patient tumor samples. In addition, we show that *HOXA9* promoter methylation status might be used as a potential biomarker for predicting cisplatin sensitivity in patients with MIBC. Furthermore, we found that low-dose decitabine and vorinostat treatment was able to induce sensitization to cisplatin and other common chemotherapeutic agents in resistant cell lines. 

## 2. Results

### 2.1. Bladder Cancer Cell Lines Have a Distinct Resistance Pattern to Chemotherapeutic Drugs

**Figure 1:**
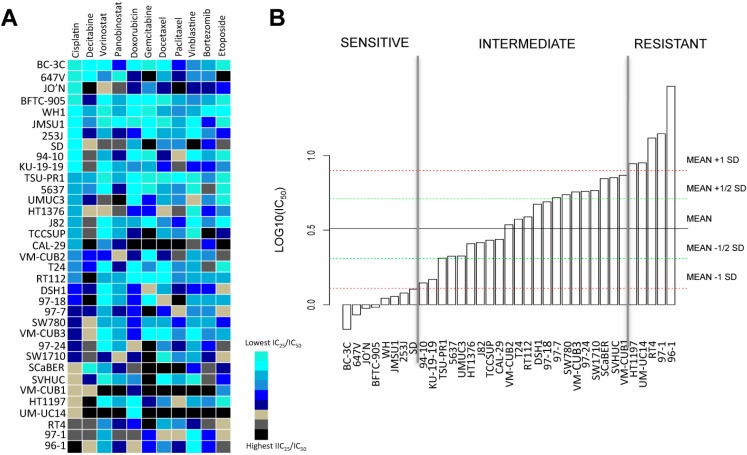
Drug screening reveals sensitivity and resistance of 35 bladder cancer cell lines to decitabine and standard chemotherapeutic agents with effects in bladder cancer. (**A**) Distinct 25%/50% inhibitory concentration (IC_25_/IC_50_) values for cisplatin and chemotherapy drugs are observed in the panel of 35 bladder cancer cell lines treated for 48 h with the respective agents. Cell lines are ranked (from lowest to highest, from top to bottom) on the basis of IC_25_/IC_50_ values for cisplatin. Color scale is normalized for each drug. Note that there is no correlation between resistance towards cisplatin and resistance towards decitabine (5-AZA-CdR); (**B**) Bladder cancer cell lines segregate into sensitive, intermediate and resistant groups according to their sensitivity to cisplatin. Sensitive cell lines: IC_50_ < average (IC_50_) − 1 SD (standard deviation); resistant cell lines: IC_50_ > average (IC_50_) + 1 SD. Experiments were run in triplicates to obtain mean IC_50_ values.

We assembled a panel of 35 bladder cancer cell lines of all stages and grades (see Table S1) and analyzed their sensitivity to cisplatin. We performed similar analyses with other chemotherapeutics such as doxorubicin, gemcitabine, docetaxel, paclitaxel, vinblastine, bortezomib, and etoposide, as well as the epigenetic modifiers decitabine, vorinostat and panobinostat. Sensitivity was determined by exposing each cell line to a series of 11 concentrations of the respective drugs and calculating the 25% inhibitory concentration/50% inhibitory concentration (IC_25_/IC_50_) values for each of them. Distinct patterns of resistance to cisplatin, vorinostat and decitabine were observed among cell lines (Figure 1A). Every agent including cisplatin harbored a different pattern of sensitivity in the cell lines. 

We next ranked cell lines based on the half maximal inhibitory concentration (IC_50_) of cisplatin. Based on these IC_50_ ranks, we assembled the cell lines into three categories, which corresponded to high, intermediate, and low cisplatin resistance (Figure 1B). Four cell lines, BC3C, 647V, JO’N and BFTC905, showed high cisplatin sensitivity (IC_50_ < average (IC_50_) − 1 SD (standard deviation)), whereas UMUC14, RT4, 96-1 and 97-1 showed low cisplatin sensitivity (IC_50_ > average (IC_50_) + 1 SD). There was no association between the original stages/grades of the patients’ tumors from which the cell lines derive and the resistance patterns (see Table S1). 

### 2.2. A Specific Gene Expression Signature is Associated with Cisplatin Resistance

**Figure 2:**
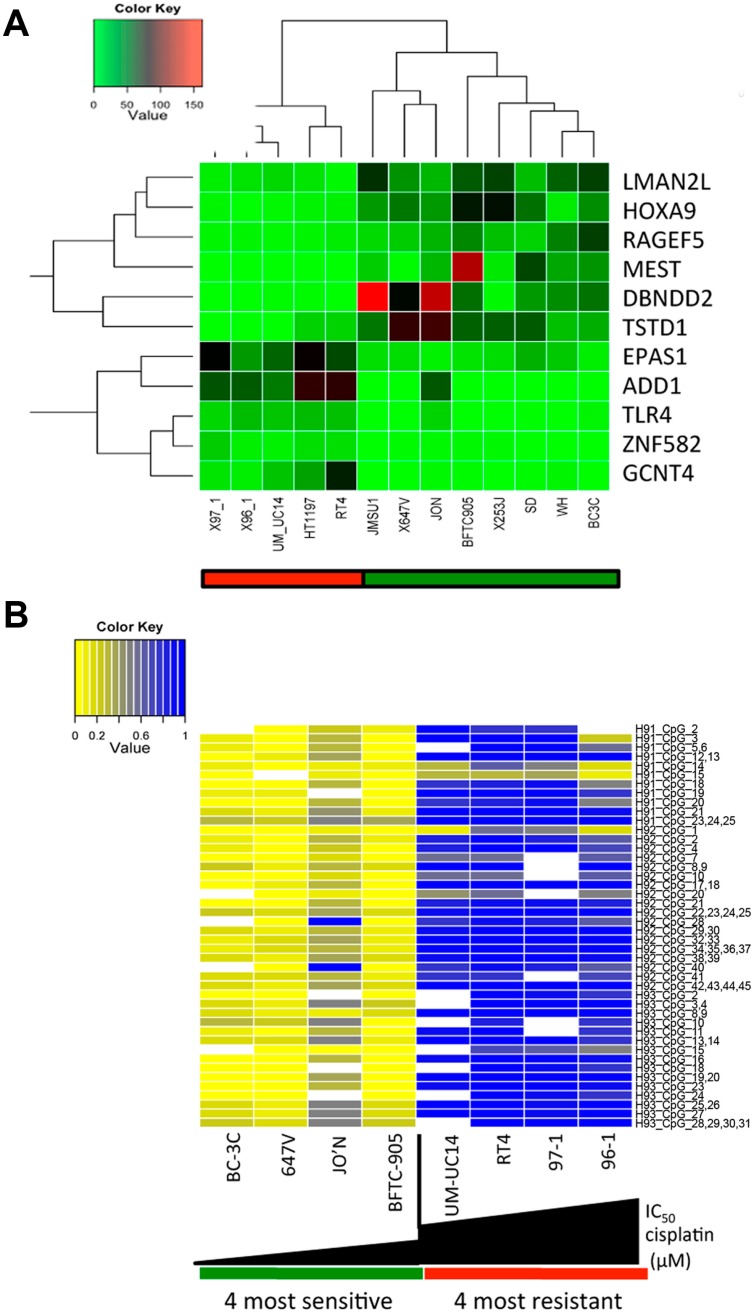
*HOXA9* promoter methylation as marker for sensitivity and resistance in bladder cancer cell lines. (**A**) Expression profiling and hierarchical clustering of top sensitive and top resistant bladder cancer cell lines identifies candidate genes to explain sensitivity and resistance to chemotherapy. The LIMMA package (version 3.28.20) was used for analyzing differential expression of RNA sequencing (RNAseq) data between sensitive and resistant cell lines; (**B**) The *HOXA9* promoter is methylated in resistant cell lines (*p* < 0.001). Methylation quantification of the *HOX9A* promoter was carried out using the EpiTYPER assay.

To assess whether specific gene expression signatures predict cisplatin sensitivity and resistance, we performed transcriptome analysis of all 35 cell lines by RNA sequencing (RNAseq) to identify genes that were differentially expressed between our cisplatin resistant and sensitive cell lines (see Figure 1B). Our analysis revealed that nine genes (*HOXA9*, *RAPGEF5*, *DBNDD2*, *TSTD1*, *EPAS1*, *ADD1*, *TLR4*, *ZNF582* and *GCNT4*) were differentially expressed between these two groups (Figure 2A). *HOXA9*, *RAPGEF5*, *DBNDD2* and *TSTD1* were downregulated, whereas *EPAS1*, *ADD1*, *TLR4*, *ZNF582* and *GCNT4* were upregulated in cisplatin resistant cell lines (see Table S2). 

We were especially interested in the potential application of *HOXA9* as a biomarker, as promoter methylation of *HOXA9* has been associated with aggressive tumor features, such as more advanced tumor stage, higher grade, larger tumor size and worse prognosis in patients with non-muscle-invasive bladder cancer [12,13,14]. The lower expression of *HOXA9* suggested that its expression could be silenced by DNA methylation at its promoter region in resistant cell lines, which would allow for the establishment of a DNA methylation-based biomarker with several potential advantages over gene expression markers [15]. 

### 2.3. *HOXA9* Promoter Methylation Levels Are Associated with Cisplatin Resistance in Bladder Cancer Cell Lines

*HOXA9* methylation has been proposed as a urinary biomarker for bladder cancer diagnosis [16]. Based on this and our own findings, we therefore confirmed *HOXA9* promoter methylation in resistant cell lines. The *HOXA9* methylation levels of the CpG islands in each of the cell lines of the sensitive and resistant groups were quantified by real-time quantitative methylation-specific polymerase chain reaction (Figure 2B). The pattern of methylation of *HOXA9* was significantly different between resistant and sensitive cell lines, with resistant cell lines exhibiting the highest levels of methylation (*p* < 0.001).

### 2.4. *HOXA9* Promoter Methylation Levels Are Associated with Response to Neoadjuvant Cisplatin-Based Combination Chemotherapy in Patients with MIBC

**Figure 3:**
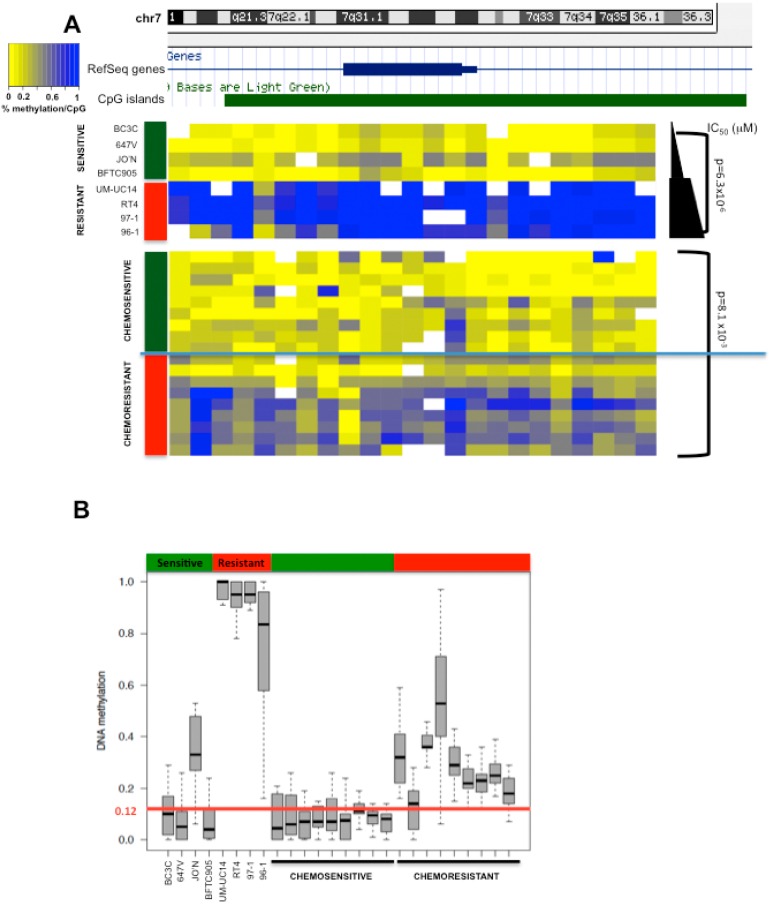
*HOXA9* promoter methylation is associated with chemoresistance of human muscle-invasive bladder cancer tissue. (**A**) CpG methylation at the *HOXA9* promoter is increased in resistant compared to sensitive tumor samples. Methylation quantification of tumor samples was carried out using the EpiTYPER assay; (**B**) Based on methylation levels detected in the cisplatin sensitive cell lines, a cut-off methylation level of 12% was defined as predictive of cisplatin resistance. Resistant tumor samples (*n* = 9) show methylation levels >12% (*p* < 0.001).

Next, we aimed to further confirm these findings in selected patients with MIBC responding or not responding to chemotherapy. We confirmed the differential pattern of *HOXA9* methylation between cisplatin resistant and sensitive tumors (Figure 3A,B). The resistant tumors (pT3–pT4) exhibited significantly higher levels of methylation of *HOXA9* when compared to sensitive tumors (pT0) (*p* < 0.001). Moreover, the cut-off of 12% was accurate in all cases.

### 2.5. Decitabine Administration Reduces Cisplatin Dose Application and Induces in Vitro Sensitization of Initially Resistant Bladder Cancer Cell Lines

We hypothesized that the DNMT inhibitor decitabine (5-aza-deoxycytidine, 5-AZA-CdR) or the HDAC inhibitor vorinostat (suberanilohydroxamic acid, SAHA) might be able to enhance sensitivity to cisplatin in resistant cell lines. This hypothesis was based on previous evidence showing that inhibitor-induced changes in epigenetic patterns are able to return resistant tumor cells to a baseline state of treatment susceptibility [6,10]. 

**Figure 4:**
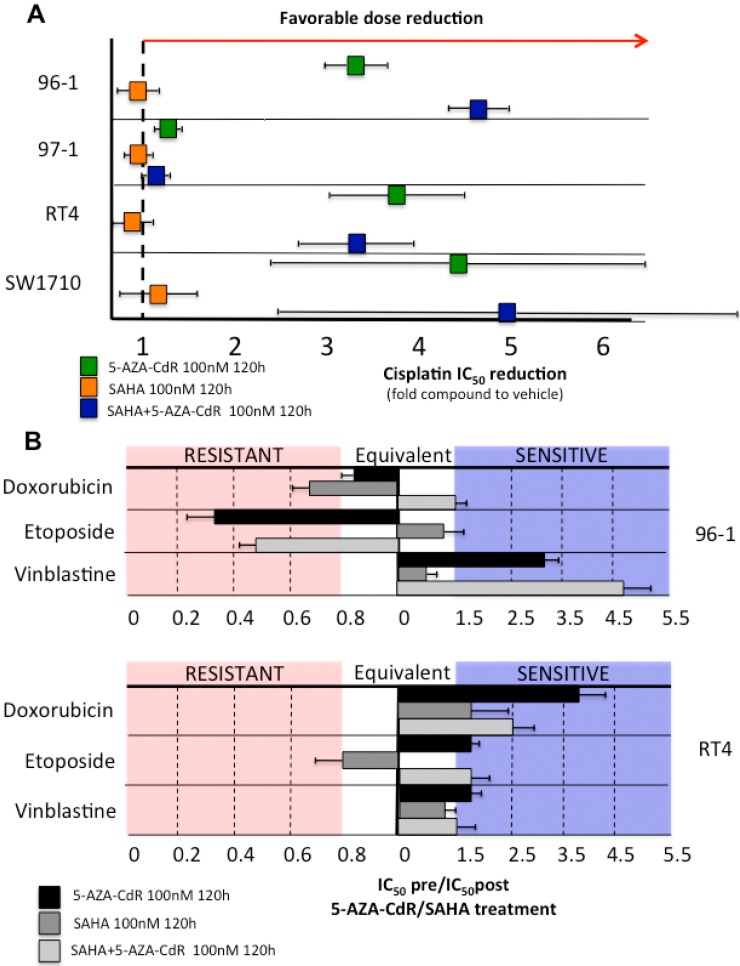
Chemoresistant bladder cancer cell lines can be sensitized to chemotherapeutics by epigenetic modifiers. (**A**) Treatment with the epigenetic inhibitors decitabine and vorinostat significantly lowers IC_50_ values for cisplatin in the resistant bladder cancer cell lines 96-1, 97-1, RT4 and SW1710. Cell lines were treated with 100 nM decitabine, 100 nM vorinostat, 100 nM decitabine + vorinostat or vehicle (DMSO) for 120 h, incubated with cisplatin for 48 h and then cisplatin IC_50_ values were calculated. The graph shows fold compound reduction to vehicle. Experiments were run in triplicates to obtain mean IC_50_ values; (**B**) Decitabine and vorinostat induce sensitization for doxorubicin, etoposide and vinblastine in the cisplatin resistant bladder cancer cell lines 96-1 (top) and RT4 (bottom). Cell lines were treated with 100 nM decitabine, 100 nM vorinostat, 100 nM decitabine + vorinostat or vehicle for 120 h, incubated with cisplatin for 48 h and then cisplatin IC_50_ values were calculated. The graph shows IC_50(vehicle)_/IC_50(post-AZA)_. Experiments were run in triplicates to obtain mean IC_50_ values. 5-AZA-CdR: decitabine; SAHA: vorinostat.

To address this, we evaluated whether pre-treatment of the top resistant (96-1, 97-1, RT4) cell lines and an intermediate (SW1710) cell line with the two epigenetic inhibitors alone and in combination at non-toxic doses (100 nM) has an effect on cisplatin sensitivity. We first confirmed that a concentration of 100 nM applied over 120 h (a time period comprising 3–4 population doublings with passive demethylation/acetylation due to inhibitors) does not have an effect on cell proliferation in these cell lines (Supplementary Figure S1A). We chose these cell lines, as we expected them to demonstrate a significant increase in cisplatin sensitivity after epigenetic treatment. Whereas pre-treatment with 100 nM vorinostat (SAHA) for 120 h led to no significant dose reduction in all tested cell lines, decitabine (5-AZA-CdR) induced a four- to fivefold IC_50_ reduction for cisplatin in 96-1, RT4 and also SW1710 cells compared to IC_50_ values without sensitization treatment (Figure 4A and Supplementary Figure S1B). No significant effect on cisplatin dose reduction was observed in the resistant cell line 97-1, however, 97-1 had higher IC_50_ values for decitabine compared to the other three cell lines (see Figure 1). Noteworthy, combined treatment with decitabine and vorinostat enhanced sensitivity in resistant cell lines 96-1, RT4 and SW1710, but no or only moderate effects were seen in the cisplatin resistant cell line 97-1.

We chose the two cisplatin resistant cell lines, 96-1 and RT4, that had shown the most significant increase in cisplatin sensitivity after epigenetic sensitization to test whether IC_50_ values for a selection of other common chemotherapeutics in urogenital cancers (doxorubicin, etoposide, and vinblastine) would decrease after pre-treatment with decitabine, vorinostat or a combination of both. As illustrated in Figure 4B, decitabine and combined decitabine and vorinostat treatment significantly lowered IC_50_ values for vinblastine in 96-1 cells and for doxorubicin, etoposide and vinblastine in RT4 cells. As observed for cisplatin resistance, single vorinostat pre-treatment did not have a pronounced effect on drug sensitization.

## 3. Discussion

In the present study, we found in a translational stepwise approach that promoter DNA methylation of the homeobox (HOX) gene *HOXA9* could help predict resistance or response to cisplatin-based chemotherapy in patients with bladder cancer. HOX genes belong to a group of transcription factors that regulate limb development during embryogenesis and are involved in tissue differentiation. *HOXA9* is part of the A cluster on chromosome 7, and has been shown to act as an oncogene in acute myeloid leukemia, whereas silencing of *HOXA9* by promoter methylation has been detected in several solid tumors such as breast, lung, ovarian and oral cancer [17,18,19,20,21]. In breast cancer, *HOXA9* was reported to regulate the expression of the tumor suppressor *BRCA1*, and re-expression of *HOXA9* led to reduced proliferation and migration of malignant cells [22]. In bladder cancer, promoter methylation of *HOXA9* has been associated with detection, aggressiveness and prognostication [12,13,14]. Here, we first report its potential application as a candidate predictive biomarker for patients receiving neoadjuvant cisplatin-based chemotherapy. However, due to low frequency of neoadjuvant chemotherapy in MIBC patients, small patient sample size is a major limitation in our patient study, and future validation comprising larger and more comprehensive patient sets will be needed. Additionally, another limitation is that this study focused on the identification of potential predictive biomarkers for neoadjuvant chemotherapy, but did not aim to investigate the molecular role of *HOXA9* in this disease. In general, silencing of HOX genes by DNA methylation has been detected in a range of cancer types and has been associated with an epigenetic stem cell signature. In this cancer-specific signature, DNA methylation replaces repressive histone methylation marks found at genes important for development and differentiation in embryonic stem cells [23,24]. Thus, it might be possible that resistant cell lines show a more “stem cell”-like epigenome, which could be altered by decitabine treatment. 

We also have demonstrated that sensitization of cisplatin resistant bladder cancer cell lines can be achieved in vitro by pre-treatment using the Food and Drug Administration (FDA)-approved DNMT inhibitor decitabine alone and in combination with the HDAC inhibitor vorinostat, confirming a previous in vitro finding on cisplatin and decitabine co-treatment in bladder cancer cells by Shang et al. [25]. Thus, decitabine treatment could potentially be used for sensitization of cisplatin-resistant bladder cancer in patients. Besides sensitization of resistant cells, it is noteworthy to mention that decitabine treatment might also lead to re-expression of so-called “cancer-testis” antigens in tumor cells of patients, which would represent a target for antibodies and open up immunotherapy approaches [26,27,28]. Combination of platin-based chemotherapy with decitabine will require correct dosing in patients, as dosing schedules are important for optimal treatment efficacy [29]. Successful sensitization of platinum resistant cancer by decitabine has been observed in diffuse B-cell lymphoma, ovarian cancer and lung cancer, and a phase 1 clinical study using azacytidine for sensitization and comprising a cohort of platinum-refractory metastatic bladder cancer patients has completed recruitment (NCT01478685) [9,10,11]. However, lack of efficacy regarding combination of platin-based chemotherapy with decitabine in ovarian cancer has also been reported, indicating that patient stratification (based on determination of *HOXA9* methylation levels in tumors) might be necessary for successful outcome in bladder cancer patients [9,10,11,30]. Sufficient sensitivity to decitabine itself will also be necessary, which is supported by our in vitro sensitization data with the cell line 97-1. 97-1 showed a significantly larger IC_50_ value for decitabine compared to cell lines 96-1, RT4 and SW1710, and could—in contrast to the other three—not be sensitized by the DNA methylation inhibitor. After validation of these findings, eligible patients with low *HOXA9* methylation levels could be offered cisplatin-based combination chemotherapy, while those with high levels will be recommended to undergo immediate radical cystectomy. Alternatively, patients with high levels of *HOXA9* methylation could receive several days of low doses of decitabine before chemotherapy for sensitization of chemoresistant tumors. Furthermore, *HOXA9* promoter methylation could be used as read-out for treatment response as a monitoring biomarker [31]. In order to translate these in vitro findings to the clinics, one could envision a neoadjuvant clinical trial of decitabine prior to cisplatin-based chemotherapy in patients with metastatic bladder cancer and integrate *HOXA9* promoter methylation, in a correlative biomarker experiment, to assess its association with treatment outcomes.

## 4. Materials and Methods

### 4.1. Cell Line Collection

Bladder cancer cell lines 5637, HT1197, HT1376, J82, RT4, SCaBER, SW780, T24, TCCSUP and UM-UC3 were purchased from ATCC (Manassas, VA, USA). 647V, BC-3C, BFTC-905, CAL-29, JMSU-1, KU-19-19, RT-112, SW1710 and VM-CUB1 were purchased from DSMZ (Riverdale, MD, USA). 253J, SV-HUC, TSU-PR1, UM-UC14 and WH were obtained from Dr. Jer-Tsong Hsieh (University of Texas Southwestern, Dallas, TX, USA). Bladder cell lines 94-10, 96-1, 97-1, 97-7, 97-18, 97-24, DSH1, JO’N, SD, VM-CUB2 and VM-CUB3 were obtained from Dr. Margaret A. Knowles (University of Leeds, Leeds, UK). Cell lines were re-authenticated via short tandem repeat (STR) analysis using the Cell-ID-system (G9500, Promega, Nacka, Sweden), and products analyzed using an Applied Biosystems 3130 Genetic Analyzer (San Francisco, CA, USA). Cell lines were cultured according to the manufacturer recommendations. We performed tests for *Mycoplasma sp*. and cell identification by Single Nucleotide Polymorphism (SNP) using the MycoSEQ detection kit (Applied Biosystems, Waltham, MA, USA).

### 4.2. Cell Lines and Drugs

Doxorubicin (Sigma, St. Louis, USA), 5-aza-2′-deoxycytidine (decitabine/5-AZA-CdR, Sigma), azacitidine (Celgene, Summit, NJ, USA), cisplatin (Sigma), gemcitabine (Sigma), etoposide (Sigma), docetaxel (Sigma), paclitaxel (Sigma), vinblastine (Sigma), panobinostat (Sigma), vorinostat (Sigma) and bortezomib (Sigma) were added from a stock solution to the 10% serum-containing RPMI medium (Gibco, Waltham, MA, USA). Doxorubicin, etoposide, docetaxel, paclitaxel, vinblastine, panobinostat, vorinostat and bortezomib were dissolved in dimethyl sulfoxide (DMSO; Sigma), cisplatin was dissolved in 10% DMSO (Sigma) in 10% serum-containing RPMI, and 5-AZA-CdR, azacitidine and gemcitabine were dissolved in distilled water. Dose calculation was based on human dosages and previous publications [10].

### 4.3. IC_50_ Determination

Cell viability was determined using a fluorometric resazurin reduction method (CellTiter-Blue, Promega) and trypan blue automatic method (TC10, Bio-Rad, Philadelphia, PA, USA). Fluorescence (Ex560 nm/Em590 nm) was determined with the Synergy4 microplate reader (BioTek, Vinooski, VT, USA). The number of viable cells was calculated by using the linear least-squares regression of the standard curve. Fluorescence was determined for three replicates and normalized to respective controls. To plot dose–effect curves CompuSyn (Biosoft, Cambridge, UK) and GraphPad Prism software 6.0 (GraphPad Prism, San Diego, CA, USA) were used, and drug concentrations inhibiting the growth of the cell lines by 50% compared to control (IC_50_) were determined. Data are presented as the mean IC_50_ or IC_25_ with a 95% confidence interval for triplicate experiments.

### 4.4. Cisplatin Sensitivity

To determine phenotypes of sensitivity to cisplatin, we exposed cells to a range of 11 concentrations of cisplatin (0.0025–150 μM) (EMD Chemicals, Inc., San Diego, CA, USA). Cell lines were categorized based on the IC_50_. The 35 cell lines were classified into two groups: one group of extreme sensitivity (average (IC_50_) − one SD (IC_50_)) and one group of extreme resistance to cisplatin (average (IC_50_) + one SD (IC_50_)). Results were aligned by the Z-score method.

### 4.5. RNA Extraction and Sequencing

Using the PureLink RNA mini kit (Invitrogen, Waltham, MA, USA), we extracted RNA from the cell lines. We used TruSeq RNA Library Preparation Kit (Illumina, San Diego, CA, USA) to make sequening libraries. RNA was sequenced using an Illumina HiSeq platform (Weill Cornell Medical College Genomics Resources Core Facility, New York, NY, USA). We obtained 60–70 million paired-end reads (51bp × 2) per cell line. After alignment using TopHat (version 1.2.0, Johns Hopkins University, USA), we estimated transcript levels using CuffLinks (version 0.9.3, University of Washington, USA) with upper-quartile and GC content normalization.

### 4.6. Sensitization of Cell Lines by Epigenetic Drugs

We exposed 97-1, SW1710, RT4 and 96-1 cells to decitabine at 100 nmol/L for 120 h with or without vorinostat at the same dose and then treated the cell lines with a range of ten different concentrations of cisplatin (0.3–3000 μM), etoposide, vinblastine or doxorubicin (0.1–1000 nM). IC_50_ values for cisplatin, etoposide, vinblastine or doxorubicin were measured after 48 h of exposure and compared to IC_50_ values without sensitization treatment using the method described above. Experiments were performed in triplicates for each drug.

### 4.7. DNA Methylation Assays

Total genomic DNA was extracted from eight bladder cancer cells using the PureLink mini kit (Invitrogen). EpiTYPER assays (Sequenom, San Diego, CA, USA) were performed on bisulfite-converted DNA. Bisulfite conversion was performed using the EZ DNA Methylation kit from Zymo Research (Irvine, San Diego, CA, USA). EpiTYPER (Sequenom) primers were designed to cover CpG islands associated with the respective HpaII amplifiable fragments. All primers were designed using Sequenom EpiDesigner beta software. Primer sequences are shown in Table S3.

### 4.8. Patient Selection and Pathological Analysis

The study population included 18 patients who were identified retrospectively in a departmental cystectomy database (Weill Cornell Medical College, Department of Urology, New York, NY, USA) and had received a complete course of neoadjuvant cisplatin-based combination chemotherapy before radical cystectomy in the time from November 2006 to November 2010. The 15 males and three females had a mean age of 69 years at the time of cystectomy (median 71, range 60 to 77). All had histologically confirmed T2 or higher MIBC tumors in previous transurethral resection. Neoadjuvant chemotherapy consisted of the standard gemcitabine–cisplatin regimen administered for 3–4 cycles. Response to chemotherapy was defined histopathologically as the absence of residual tumor (pT0 stage) on the radical cystectomy specimen and resistance to cisplatin was defined as locally advanced disease (stage pT3–pT4). Bladder specimens from transurethral resection for DNA isolation were obtained after approval by the institutional review board and informed consent.

### 4.9. DNA Extraction from Tumor Tissue of Patients with Muscle-Invasive Bladder Cancer

Tissue DNA was extracted using the DNA Purification Kit (Qiagen, Valencia, CA, USA). One microgram of DNA was bisulfite-modified using EpiTect (Qiagen) for the EpiTYPER assay and DNA methylation levels were determined as described above for cell lines. 

### 4.10. Statistics and Bioinformatics

Analysis was performed using the R open source Statistical software version 2.11.1 and Prism (GraphPad Software, version 6.0). To determine the differentially expressed and methylated genes we computed a *t*-test or LIMMA on normalized arrays (after filtering for genes with a standard deviation greater than 0.5). R Bioconductor LIMMA Voom package was used (voom, lmFit, and eBayes functions) (Bioconductor, version 3.28.20). We filtered by adjusted *p* value.

Methylation differences were evaluated by a non-parametric Wilcoxon–Mann–Whitney-test (Stata 11, Statacorp, College Station, TX, USA). A cut-off methylation level of 12% was defined as predictive of cisplatin resistance.

## Supplementary Materials

The following are available online at http://www.mdpi.com/2218-273X/6/3/37/s1. Figure S1: Effect of low-dose epigenetic inhibitor treatment on cell proliferation and half maximal inhibitory concentration (IC_50_) curves for cisplatin, Table S1: Bladder cancer cell lines including known stages, grades and origins used in this study, Table S2: List of top differentially expressed genes between cisplatin-resistant and cisplatin-sensitive bladder cancer cell lines, Table S3: Primers and probes for *HOXA9* promoter methylation analysis using the EpiTYPER assay.
