# Supplementary material for: An Epigenomic Approach to Improving Response to Neoadjuvant Cisplatin Chemotherapy in Bladder Cancer
Source: Biomolecules. 2016 Sep 2;6(3):37. doi: 10.3390/biom6030037 (PMC5039423; doi:10.3390/biom6030037)
Supplement: Supplementary file 1 [file biomolecules-06-00037-s001.docx]

Supplementary Materials: An Epigenomic Approach to Improving Response to Neoadjuvant Cisplatin Chemotherapy in Bladder Cancer

Evanguelos Xylinas, Melanie R. Hassler, Dazhong Zhuang, Martin Krzywinski, Zeynep Erdem, Brian D. Robinson, Olivier Elemento, Thomas Clozel and Shahrokh F. Shariat

**Figure S1.** Effect of low-dose epigenetic inhibitor treatment on cell proliferation and half maximal inhibitory concentration (IC_50_) curves for cisplatin. (**A**) Treatment with the epigenetic inhibitors decitabine and vorinostat at 100 nM for 120 h does not have an effect on cell proliferation or viability in the bladder cancer cell lines 96-1, 97-1, RT4 and SW1710. Cell lines were seeded at 1 × 10^5^ cells/mL, treated with 100 nM decitabine, 100 nM vorinostat, 100 nM decitabine + vorinostat or vehicle for 120 h and then cells were counted. No significant difference was detected among treatment regimens for individual cell lines. Groups were analysed by one-way analysis of variance; (**B**) IC_50_ curves for cisplatin in cell lines 96-1, 97-1, RT4 and SW1710. Cell lines were treated with 100 nM decitabine, 100 nM vorinostat, 100 nM decitabine + vorinostat or vehicle for 120 h and then cells were incubated with varying concentrations of cisplatin to obtain IC_50_ curves. The graph shows mean values ± standard deviation. Experiments were performed in triplicates to obtain mean IC_50_ values. PBS: phosphate-buffered saline; AZA/5-AZA-CdR: decitabine; SAHA: vorinostat.

**Table S1.** Bladder cancer cell lines including known stages, grades and origins used in this study.

| **Number** | **Cell Line** | **Culture Medium** | **In Vitro Morphological Characteristics** | **Grade** | **Stage** | **Origination** |
| --- | --- | --- | --- | --- | --- | --- |
| 1 | 253J | RPMI + 10%FBS | TCC look-like | G4 | T4 | LN metastasis |
| 2 | 5637 | RPMI + 10%FBS | epithelial-morphology, small cells |  |  | Bladder tumor |
| 3 | 639-V | RPMI + 10%FBS |  | G3 |  | Ureter |
| 4 | 647-V | RPMI + 10%FBS | small, epithelial cells, multilayer | G2 |  | Bladder tumor |
| 5 | 94-10 | RPMI + 10%FBS |  | G2-3 | T3 | Bladder tumor |
| 6 | 96-1 | RPMI + 10%FBS |  | G2-3 | T3 | Bladder tumor |
| 7 | 97-1 | RPMI + 10%FBS |  | G1 | T1/2 | Bladder tumor |
| 8 | 97-18 | RPMI + 10%FBS |  | G3 | T2 | Bladder tumor |
| 9 | 97-24 | RPMI + 10%FBS |  | G3 | T3 | Bladder tumor |
| 10 | 97-7 | RPMI + 10%FBS |  | G2-3 | T1 | Bladder tumor |
| 11 | BC-3C | RPMI + 10%FBS |  | G4 |  | Bladder tumor |
| 12 | BFTC-905 | RPMI + 10%FBS |  | G3 |  | Bladder tumor |
| 13 | CAL-29 | RPMI + 10%FBS |  |  |  |  |
| 14 | HT1197 | RPMI + 10%FBS |  |  |  | Bladder tumor |
| 15 | HT1376 | RPMI + 10%FBS | moderately pleomorphic | G3 | >T2 | Bladder tumor |
| 16 | J82 | RPMI + 10%FBS | mixed epithelial, fibroblastic | G3 | T3 | Bladder tumor |
| 17 | JMSU-1 | RPMI + 10%FBS |  |  |  | Bladder tumor |
| 18 | J'ON | RPMI + 10%FBS |  |  |  | Bladder tumor |
| 19 | KU-19-19 | RPMI + 10%FBS |  | G3 | T3b | Bladder tumor |
| 20 | RT-112 | RPMI + 10%FBS |  | G2 |  | Bladder tumor |
| 21 | RT4 | RPMI + 10%FBS | well differentiated transitional cells | G1-2 | T2 | Bladder tumor |
| 22 | SCaBER | RPMI + 10%FBS |  | G2 | T3 | Squamous cell carcinoma bladder |
| 23 | SV-HUC-1 | RPMI + 10%FBS |  |  |  |  |
| 24 | SW1710 | RPMI + 10%FBS | large polygonal cells | G3 | Ta | Bladder tumor |
| 25 | SW780 | RPMI + 10%FBS | round or ovoid cells | G1 | T1 | Bladder tumor |
| 26 | T24 | RPMI + 10%FBS | epidermoid TCC cells | G3 |  | Bladder tumor |
| 27 | TCCSUP | RPMI + 10%FBS |  | G4 | >T2 | Bladder tumor |
| 28 | TSU-PRI | RPMI + 10%FBS |  |  |  |  |
| 29 | UM-UC14 | RPMI + 10%FBS |  |  |  |  |
| 30 | UM-UC3 | RPMI + 10%FBS | undifferentiated, spindle-shaped cells |  |  | Bladder tumor |
| 31 | VM-CUB1 | RPMI + 10%FBS |  |  |  | Bladder tumor |
| 32 | VMCUB2 | RPMI + 10%FBS |  |  |  | LN metastasis |
| 33 | VMCUB3 | RPMI + 10%FBS | TCC cells |  |  | Bladder tumor |
| 34 | WH | RPMI + 10%FBS |  |  |  |  |

RPMI: Roswell Park Memorial Institute medium; FBS: fetal bovine serum; TCC: transitional cell carcinoma; LN: lymph node (retroperitoneal)

**Table S2.** List of top differentially expressed genes between cisplatin-resistant and cisplatin-sensitive bladder cancer cell lines.

| **Gene** | **Full Name/Protein** | **Function** | **Expression (Resistant^1^)** |
| --- | --- | --- | --- |
| *HOXA9* | Homeobox protein | transcription factor | down |
| *RAPGEF5* | Rap guanine nucleotide exchange factor 5 | Ras activator | down |
| *DBNDD2* | Dysbindin domain-containing protein 2 | casein kinase-1 binding protein | down |
| *TSTD1* | Thiosulfate sulfurtransferase | sulfurtransferase | down |
| *EPAS1* | Endothelial PAS domain-containing protein 1 | hypoxia-inducible factor | up |
| *ADD1* | Alpha-adducin | cytoskeleton | up |
| *TLR4* | Toll-like receptor 4 | innate immune system activation | up |
| *ZNF582* | Zinc finger protein 582 | zinc finger protein | up |
| *GCNT4* | Glucosaminyl (*N*-acetyl) transferase 4 | glycosylation | up |

**^1^** Up/down-regulated in resistant compared to sensitive cell lines.

**Table S3.** Primers and probes for *HOXA9* promoter methylation analysis using the EpiTYPER assay.

| **Amplicon Name** | **Target Length** | **Number of CpGs** | **L^1^/ R^2^** | **Target Sequence** |
| --- | --- | --- | --- | --- |
| HOXA9_000 | 465 | 25 | aggaagagagTGAAATTTGGAAATAGGTTTTTTTG/  cagtaatacgactcactatagggagaaggctCTTAACTACCCAAACCCAAAACC | TGAAATTTGGAAACAGGTCTCCTTGCCACGCAGAGGGAAAGCATGCTGCCTTGTGTTCTGTAGCAAGATTAGGATTCCTCTGTCCCGTTCACTGACTTCGTCTTTCTTTCCCAACCTGTCCCTCTACGCCCCCCACTCCTTATTTAACCTTCCTGGAAGGCCTTCGGAGCTGGGCAAGCCGTCAGGGCGCCCTAAGGCCGCTGATCACGTCTGTGGCTTATTTGAATAATCTGTCATGGGGACCCTTGTGGCCCGGGTCGCCCGCAGCCTCATCTTGGCAGGATTTACGCCGCCACTGGCCGAAGGCAAGAAGTGGAAGGAATCGGCCGTCTCCCCCAGCGTCCCAGCTCCGGCTGCCCTGGCTGCCGCCGCTCACGGACAATCTAGTTGTACAAAAGGCTCTCTGGGCTGCACTGCTTTCGAAGAACGGCCCAAAGTATCTCGGTCCTGGGCCTGGGCAGCCAAG |
| HOXA9_0001 | 488 | 30 | aggaagagagAAGGTTTTTTGGGTTGTATTGTTTT/  cagtaatacgactcactatagggagaaggctAACACTCACACTTTATCCCTAACTAA | AAGGCTCTCTGGGCTGCACTGCTTTCGAAGAACGGCCCAAAGTATCTCGGTCCTGGGCCTGGGCAGCCAAGGAGAGGGGCGGCCAGTCTTGGCTCGTCCCGAAGTGCCCGCCCCGCCCCCTCTCGCTGCAGCAGCCGCCTCCTCTCCCGTAGCCCTGCGGGCCGCTCTTCACTGCTCTCCAGACTTGGGGCCCTATCTGAGGCGTCCCAAACACCAACTTCTGGCTCCTGGCCCCAACTCGAGAGGCTTCCAGCGAGGACGAAGGCAGGCTCGAGAGAAACCTGGCGGGCCAGCAGATCCGGGAGGCCGGCGTGGAGGCGGCGGCGGATTTGAAGGGAGGAGACACTTACTGGGATCGATGGGGGGCTTGTCTCCGCCGCTCTCATTCTCAGCATTGTTTTCAGAGAAGGCGCCTTCGCTGGGTTGTTTTTCTCTATCAACTGGAGGAGAACCACAAGCATAGTCAGTCAGGGACAAAGTGTGAGTGTC |
| HOXA9_0002 | 498 | 48 | aggaagagagTTTTTTTTATTAATTGGAGGAGAAT/  cagtaatacgactcactatagggagaaggctCCACCACTAAAACCCTAAACAACTA | TTTTCTCTATCAACTGGAGGAGAACCACAAGCATAGTCAGTCAGGGACAAAGTGTGAGTGTCAAGCGTGGGACAGTCACCCCTTCTGGCCGACAGCGGTTCAGGTTTAATGCCATAAGGCCGGCTGGAGGGCAAGCCCGCGAAGGAGAGCGCACCGGGCGTGGGCTCCAGCCAGGAGCGCATGTACCTGCCGTCCGGCGCCGCCGCCGCCACGGGCGCCTGGGGGTGCACGTAGGGGTGGTGGTGATGGTGGTGGTACACCGCAGCGGGTACAGCGTTGGCGCCCGCCGCGTGCACTGGGTTCCACGAGGCGCCAAACACCGTCGCCTTGGACTGGAAGCTGCACGGGCTGAAGTCGGGGTGCTCGGCCAGCGTCGCCGCCTGCCGGGGAGGCTGGCCCAGGGTCCCCGGCGCATAGCGGCCAACGCTCAGCTCATCCGCGGCGTCGGCGCCCAGCAGGAACGAGTCCACGTAGTAGTTGCCCAGGGCCCCAGTGGTGG |
| HOXA9_0003 | 491 | 31 | aggaagagagGTTTGTGTGGTTTTTGAAATAATAA/  cagtaatacgactcactatagggagaaggctAAAAAAATACAATCACCTAATAAATTACC | GCCTGTGTGGCTTCTGAAACAATAACTCCTTATGAAATATCATAAATATAGATTTAAATACAGTAGAGCGACAATGCGATTTGGCTGCTTTTTTATGGCTTCAATTATTGTCTAATTTTATGTGAGGGGCTCCGCTGGCCGCACTCGCACGCGGGACCCGCGCCTTCTTGATGGCGTGATTAATTGTGATATAAAATAGTCCGCTTAAGAAGTGTGTGTATGGGGGGGGAGACGGGAGAGTACAGAGACAAGGCTAGATTTGATCTTTTAATCGTCGTTGGCCACAATTAAAACAAACCCCATCGTAGAGCGGCACGATCCCTTTACATAAAAACATATGGCTTTTGCTATAAAAATTATGACTGCAAAACATCGGACCATTAATAGCGTGCGGAGTGATTTACGCGTTATTGTTCTGCTGGACGGGCACGTGACGCGCACGGCCAATGGGGGCGCGGGCGCCGGCAACTTATTAGGTGACTGTACTTCCCC |
| HOXA9_0004 | 271 | 28 | aggaagagagAGAGTTTTTGAGGTTGGAGTATAGG/  cagtaatacgactcactatagggagaaggctCCTACCAAAACACTCCAAACAAA | AGAGCCTCTGAGGCTGGAGCACAGGGCTGGGTCGCCAGCCGCCTGCGCCTGGGAATCCTGATTGCCAGCTGATGAGAAAGGCGGGCTGGGCGCGCGTGTGCGTGGGGTCGAGGGCCGGGGACCGAGCGCGCCGCACAACCAACCAGGCCCTCAAAACCTTCGCCCTGGTGGCGGCTGGCCGCTCCCTCCTGGCCAGCTCCTCCGTGGGGTCCTCGTAGCAAAGGCGAATTTAAGGGTTGCCCGGGCGCCCCTCGCTCCAGGCGGGTAGCTGTGGGGACCTACACCCGCGGTACTCCCTGAGCGGCCGGTCCCTGCCTGGAGTGCCCTGGTAGG |
| HOXA9_0005 | 325 | 18 | aggaagagagGTTTTGAGTTGTGGTTGTTTTTTTT/  cagtaatacgactcactatagggagaaggctACCCTATACTCCAACCTCAAAAACT | GCCTTGAGCTGTGGCTGCCTCTCTTTGGGCCTTGTACCTCTCCGCCGAGTCTCCGGGCCCCGTAGGTAACCAAGGCGAGGCCCGGAGTAGCAGCTGGAAAGGGAGGAAGGAGCCCTGAAAGGCTCACGCGGCCCCGGGACAGGCCACATCGGTGCGGGCCTCCCAGGTTCCGGAGCTGCGGGGTCTCTTAGGCGAGGCTGCCTTTTCCCAAACCGAACTTGCCTTCCATTCATGCCACTTGTAGTTTTTTCCCCAGCTGGGATTCACGGAGCGCAACCAGGCTTGCAGCGCTCATGGTTAGAGCCTCTGAGGCTGGAGCACAGGGC |

^1^forward primer sequence, capital letters indicate start of amplicon sequence (bisulfite converted DNA)

^2^reverse primer sequence, capital letters indicate end of amplicon sequence (bisulfite converted DNA)
